# Sleep duration and risk of high blood pressure in Thai adolescents: the Thai National Health Examination Survey V, 2014 (NHES-V)

**DOI:** 10.1186/s12889-022-14430-z

**Published:** 2022-10-29

**Authors:** Kwanchai Pirojsakul, Wichai Aekplakorn, Sirinapa Siwarom, Witchuri Paksi, Pattapong Kessomboon, Nareemarn Neelapaichit, Suwat Chariyalertsak, Savitree Assanangkornchai, Surasak Taneepanichskul

**Affiliations:** 1 Department of Pediatrics, Ramathibodi Hospital Mahidol University, 270 Rama VI Road, Ratchathewi, Bangkok, 10400 Thailand; 2 Department of Community Medicine, Ramathibodi Hospital Mahidol University, Bangkok, Thailand; 3 Faculty of Medicine, Khon Kaen University, Khon Kaen, Thailand; 4 Faculty of Medicine, Ramathibodi School of Nursing, Ramathibodi Hospital, Mahidol University, Bangkok, Thailand; 5 Faculty of Public Health, Chiang Mai University, Chiang Mai, Thailand; 6 Epidemiology Unit, Faculty of Medicine, Prince of Songkla University, Songkhla, Thailand; 7 College of Public Health Sciences, Chulalongkorn University, Bangkok, Thailand

**Keywords:** Prevalence, Blood pressure, Adolescents, Sleep duration, High blood pressure, Body mass index

## Abstract

**Background:**

Sleep duration has been proposed to be associated with high blood pressure. However, nationwide studies regarding this association in adolescents remain limited. This study aimed to explore the national prevalence of high blood pressure among Thai adolescents and to determine the association between sleep duration and high blood pressure.

**Methods:**

Data from adolescents aged 10–19 years from the Thai National Health and Examination Survey V were included. We collected demographic data (including age and gender), height, weight, waist circumference, blood pressure, fasting blood chemistries and sleep duration data. Sleep durations were categorized as short, normal or long for each age group based on the United States National Sleep Foundation’s recommendations. High blood pressure was diagnosed using the 2017 guidelines of the American Academy of Pediatrics. Factors associated with high blood pressure were analyzed using multivariate logistic regression.

**Results:**

A total of 3505 adolescents (1785 female) were included. The prevalence of high blood pressure was 9.4% (95% CI 8.5–10.4%). The high blood pressure group had higher BMI z-score, LDL-C, triglyceride and lower HDL-C than the normotensive group. In the multivariate analysis, BMI z-score, LDL-C and HDL-C were independently associated with high blood pressure. However, there was no association between sleep duration and high blood pressure.

**Conclusions:**

High blood pressure risk was increased in adolescents with high BMI z-score. Neither short nor long sleep duration was associated with an increased risk of high blood pressure.

**Supplementary Information:**

The online version contains supplementary material available at 10.1186/s12889-022-14430-z.

## Background

Over the past few decades, the prevalence of elevated blood pressure in children has been rising [1]. The prevalence of elevated blood pressure reported by the National Health and Nutrition Examination Survey (NHANES) in the US increased from 15.8% to 19.2% in boys and 8.2% to 12.6% in girls during the years 1999–2008 (compared with that during the years 1988–1994) [2]. A 2015 study of school-aged children in Thailand showed that 24.3% of boys and 21.4% of girls had elevated blood pressure [3]. Obesity plays an important role as one of the causes of an increase in the prevalence of elevated blood pressure among school-aged children. One school-based study in Thailand revealed that 30.6% of boys and 12.8% of girls were obese, increasing the risk of elevated blood pressure 10.3-fold [4]. This positive association between the prevalence of obesity and hypertension in children has been reported consistently across many countries in Asia [5–8].

Apart from obesity, sleep duration has been reported to be one of the factors associated with high blood pressure in adults. However, little is known about the association between sleep duration and hypertension in adolescents. Wells et al. reported that short sleep duration was associated with increased systolic blood pressure in a cross-sectional survey of 4452 Brazilian adolescents [9]. Bal et al. found that Turkish adolescents aged 11–17 years who slept ≤ 8 h/day had a high prevalence of hypertension and pre-hypertension, up to 35% [10]. Moreover, in a national survey from Korea, adolescents who slept ≤ 5 h/day had twice the risk of elevated blood pressure compared with adolescents who slept 8–9 h/day [11]. Because the prevalence of hypertension is increasing in Thailand and most other countries, it is urgent to identify and quantify the risk factors of hypertension in adolescents and young adults to de-escalate the public health burden in the near future. In addition, to date there has been no study regarding the association between sleep duration and blood pressure in Thailand. By analyzing data from the Thai National Health and Examination Survey V, this study aimed to identify the prevalence of high blood pressure and determine whether sleep duration is associated with high blood pressure in adolescents.

## Methods

This study was conducted with approval from the Ramathibodi Hospital Ethics Committee for Human Research (MURA 2019/917). The participants included in this study were recruited from the database of the Thai National Health and Examination Survey V (NHES-V), conducted in the year 2014. The NHES-V was a nationwide cross-sectional survey using a multistage, stratified sampling of the Thai population. The method of sampling was previously described in detail [12]. In brief, the survey included 32,400 participants of all ages, starting from 1 year of age, in five regions including Bangkok, the Central region, the Northern region, the Northeastern region, and the Southern region. A total of 3559 participants aged 10–19 years were examined in the NHES-V survey. Participants without data regarding sleep duration (*N* = 54) were excluded. A total of 3505 adolescents were included in the final analysis.

Demographic and baseline data including age, weight, height, waist circumference and blood pressure were obtained. Body mass index (BMI) was calculated using the formula as follows: weight (kg)/height (m)^2^. BMI was categorized using BMI z-score based on the World Health Organization growth reference for age and gender [13]: Obesity is defined as a BMI z-score ≥ 2. Waist circumference (WC) to height ratio (WHR) was calculated as WC (cm)/height (cm). Weight was measured in kilograms (kg) to the closest one decimal place. Height was measured in centimeters (cm) to the closest one decimal place. Waist circumference was measured at the midpoint between the lower rib and the top of the iliac crest in centimeters to the closest one decimal place.

The standardized blood pressure measurement was used as recommended by the guidelines from the American Academy of Pediatrics (AAP) [14]. The blood pressure monitor used in this study was the Microlife BP 3AG1 (Widnau, Switzerland), which has been validated according to the British Society of Hypertension guidelines. Blood pressure was measured on the right arm in a sitting position after resting for 5 min. The cuff used for each participant had a width of at least 40% and the length of at least 80% of the mid-arm circumference. Each participant had three blood pressure measurements with the 5-min interval between measurements. The first measurement was discarded and the average of the last two measurements was defined as the blood pressure of each participant. Adolescents with either systolic blood pressure (SBP) or diastolic blood pressure (DBP) ≥ the 95^th^ percentile based on age, gender and height in those aged 10–12 years or ≥ 130/80 mmHg in those aged ≥ 13 years were defined as having high blood pressure [14]. The term “hypertension” was not used in this study because the blood pressure measurement was performed at only a single visit for each participant.

Data regarding the sleep–wake times for weekdays (WD) and weekends (WE) were also collected via direct interviews conducted by trained researchers. There were no questions regarding how many days the participants attended the school, but most adolescents in Thailand typically attend school 5 days a week. Therefore, average sleep duration was calculated as [(WD × 5) + (WE × 2)] / 7, as used in the previous study [15]. Recommendations from the United States National Sleep Foundation (NSF) specify that adolescents aged 10–13, 14–17 and 18–19 years should have a sleep duration of 9–11, 8–10 and 7–9 h/day, respectively [16]. Therefore, sleep duration was categorized into three groups as follows: Group 1, the group with a sleep duration less than the recommendation for their age (short sleep duration); Group 2, the group with a sleep duration within the recommendation for their age (normal sleep duration); and Group 3, the group with a sleep duration more than the recommendation for age (long sleep duration). In addition, the disparity in sleep duration between WE and WD (WE-WD) was also analyzed for each participant.

Fasting blood samples were taken for chemistry analysis including plasma glucose, total cholesterol (TC), low-density lipoprotein cholesterol (LDL-C), high-density lipoprotein cholesterol (HDL-C) and triglycerides (TG). An abnormal lipid profile was defined as LDL-C ≥ 130 mg/dl, TG ≥ 130 mg/dl or HDL-C < 40 mg/dl, according to the recommendations for adolescents from the National Heart, Lung, and Blood Institute [17].

Demographic data are presented as frequency and medians (inter-quartile ranges). The Mann–Whitney U test was used to detect differences in continuous data between two groups; the Chi-square test was used to detect the differences in categorical data between two or three groups. Multivariate analysis was used to evaluate the parameters associated with high blood pressure in two models. The independent variables in model 1 included BMI category (BMI z-score < 2 vs. each 1 z-score increase), HDL-C (> 40 vs. each 5 mg/dl decrease), LDL-C (< 130 vs. each 10 mg/dl increase), and TG (< 130 vs. each 10 mg/dl increase), and the variable of average sleep duration model was added in model 2. A *p*-value of ≤ 0.05 was considered statistically significant. Institutional licensed SPSS software version 22 was used for statistical analysis.

## Results

### Demographic data and fasting blood chemistries

A total of 3505 participants (1803 females) aged 10–19 years were included in this study. The overall prevalence of high blood pressure was 9.4% (95% CI 8.5–10.4%). Demographic and other data in the high blood pressure and the normotensive groups for all participants and separated by gender are presented in Table 1. For both genders combined, the high blood pressure group had significantly greater BMI z-score and WHR and a greater proportion of obesity than did the normotensive group. Moreover, the high blood pressure group had significantly greater median LDL-C, TG and a significantly lower median HDL-C than did the normotensive group.

**Table 1:** Characteristics of study participants

Parameters	All participants (*N* = 3505)		*P*-value	Male (*N* = 1720)		*P*-value	Female (N = 1785)		*P*-value
	**Normotension (*N* = 3176)**	**High blood pressure** **(*N* = 329)**		**Normotension (*N* = 1490)**	**High blood pressure (*N* = 230)**		**Normotension (*N* = 1686)**	**High blood pressure (*N* = 99)**	
Age (years), median (IQR)	14 (12,16.4)	13.7 (11.5,16.8)	0.363	13.9 (12,16.1)	13.6 (11.6,17.1)	0.357	14.1 (11.9,16.6)	13.7 (11.4,15.9)	0.683
Height (cm), median (IQR)	156 (148,163)	157 (147,167)	0.256	161 (148,168)	160 (147,170)	0.983	154 (148,159)	154 (147,159)	0.683
Weight (kg), median (IQR)	47 (39,56)	58 (43,77)	** < 0.001**	50 (39,59)	59 (44,80)	** < 0.001**	46 (39,53)	53 (42,68)	**0.004**
BMI (kg/m^2^), median (IQR)	19 (16.7,21.9)	23 (18,28.8)	** < 0.001**	18.8 (16.6,22)	23.5 (18.2,28.6)	** < 0.001**	19.1 (16.9,21.9)	22.1(18.1,29.3)	**0.004**
BMI z-score, median (IQR)	-0.16 (-0.83,0.89)	1.1 (-0.2,3.53)	** < 0.001**	-0.17 (-0.88,1.0)	1.2 (-0.23,3.7)	** < 0.001**	-0.16 (-0.78,0.8)	0.67 (0.02,3)	** < 0.001**
Prevalence of obesity (%)	11.89	39.82	** < 0.001**	15.03	41.74	** < 0.001**	9.02	35.35	** < 0.001**
WC (cm), median (IQR)	67 (61,75)	77 (64,92)	** < 0.001**	68 (61,76)	79 (64,93)	** < 0.001**	67 (61,74)	74 (63,88)	** < 0.001**
WHR (cm/cm), median (IQR)	0.43 (0.4,0.48)	0.48 (0.42,0.57)	** < 0.001**	0.42 (0.4,0.47)	0.49 (0.42,0.58)	** < 0.001**	0.43 (0.4,0.48)	0.47 (0.42,0.56)	** < 0.001**
SBP (mmHg), median (IQR)	105 (98,113)	127 (121,134)	** < 0.001**	109 (101,115)	129 (122,135)	** < 0.001**	103 (97,110)	125 (119,131)	** < 0.001**
DBP (mmHg), median (IQR)	63 (58,68)	77 (68,82)	** < 0.001**	63 (58,68)	76 (68,81)	** < 0.001**	63 (58,67)	79 (70,82)	** < 0.001**
^%^Glucose (mg/dl), median (IQR)	87 (83,93)	89 (83,94)	0.22	88 (83,93)	89 (84,95)	**0.015**	87 (82,93)	87 (82,92)	0.915
^@^TC (mg/dl), median (IQR)	169 (146,191)	175 (152,195)	0.2	163 (140,186)	176 (152,198)	** < 0.001**	174 (152,195)	172 (151,193)	0.541
^@^HDL-C (mg/dl), median (IQR)	50 (43,59)	46 (39,53)	** < 0.001**	48 (41,58)	46 (38,53)	**0.003**	51 (44,60)	47 (40,58)	**0.015**
^@^LDL-C (mg/dl), median (IQR)	107 (88,126)	118 (94,137)	**0.004**	104 (85,124)	117 (95,138)	** < 0.001**	111 (92,130)	112 (91,135)	0.602
^@^TG (mg/dl), median (IQR)	83 (62,111)	98 (68,132)	**0.001**	81 (60,111)	95 (67,135)	**0.002**	84 (65,111)	102 (71,126)	**0.02**
^$^WD sleep duration (hours), median (IQR)	9 (8,10)	9 (8,10)	0.828	9 (8,10)	9 (8,10)	0.483	9 (8,10)	9 (8,9.5)	0.990
^$^WE sleep duration (hours), median (IQR)	9.5 (8.5,10.2)	9.5 (8.5,10)	0.637	9.5 (8.5,10.5)	9.5 (8.5,10)	0.428	9.5 (8.5,10)	9.5 (9,10)	0.823
^$^Average sleep duration (hours), median (IQR)	9 (8.1,10)	9 (8.3,9.9)	0.983	9 (8.29,10)	9 (8.2,9.86)	0.42	9 (8,9.9)	9 (8.5,9.8)	0.583
WE-WD sleep duration (hours), median	0 (0, 1)	0 (0, 1)	0.55	0 (0, 1)	0 (0, 1)	0.638	0 (0, 1.5)	0 (0, 1.5)	0.39

*IQR* inter-quartile range, *BMI* body mass index, *WC* waist circumference, *WHR*, waist to height ratio, *SBP* systolic blood pressure, *DBP* diastolic blood pressure, *TC* total cholesterol, *HDL-C* high-density lipoprotein cholesterol, *LDL-C* low-density lipoprotein cholesterol, *TG* triglyceride, *WD* weekdays, *WE* weekends ^%^*N* = 3118, ^@^*N* = 2843, ^$^*N* = 3505, Bold text, statistical significance (*p*-value ≤ 0.05)

### Sleep duration and blood pressure

Overall, there were no significant differences in weekday, weekend and average sleep durations between the high blood pressure and normotensive groups. No differences in the disparity of sleep duration on weekdays and weekends were detected between the two groups (Table 1). There were no differences in the proportion of Group 1 (short sleep duration) or Group 3 (long sleep duration) between the high blood pressure and normotensive groups (Table 2). In addition, the numbers of participants stratified by the sleep duration groups on weekdays and weekends were also not different between the high blood pressure and normotensive groups (supplemental Table S1 and S2).

**Table 2:** Proportion of participants with and without high blood pressure categorized by the sleep duration group

Periods	Sleep duration group	All participants (*N* = 3505)		*P*-value	Males (*N* = 1720)		*P*-value	Females (*N* = 1785)		*P*-value
		**Normotension *N* = 3176**	**High blood pressure *N* = 329**		**Normotension *N* = 1490**	**High blood pressure N = 230**		**Normotension *N* = 1686**	**High blood pressure *N* = 99**	
Weekdays	1, N (%)	908 (28.59)	82 (24.92)	0.057	377 (25.3)	58 (25.22)	0.434	531 (31.44)	24 (24.24)	0.095
	2, N (%)	2024 (63.73)	230 (69.91)		993 (66.64)	159 (69.13)		1031 (61.15)	71 (71.71)	
	3, N (%)	244 (7.68)	17 (5.17)		120 (8.05)	13 (5.66)		124 (7.35)	4 (4.04)	
Weekends	1, N (%)	557 (17.54)	52 (15.81)	0.291	269 (18.05)	39 (16.96)	0.32	288 (17.08)	13 (13.13)	0.532
	2, N (%)	2025 (63.76)	224 (68.09)		933 (62.92)	155 (67.39)		1092 (64.77)	69 (69.7)	
	3, N (%)	594 (18.7)	53 (16.1)		288 (19.33)	36 (15.65)		306 (18.15)	17 (17.17)	
Average	1, N (%)	898 (28.27)	80 (24.32)	0.074	387 (25.97)	59 (25.65)	0.371	511 (30.31)	21 (21.21)	0.08
	2, N (%)	1922 (60.52)	220 (66.87)		921 (61.82)	150 (65.22)		1001 (59.37)	70 (70.7)	
	3, N (%)	356 (11.21)	29 (8.81)		182 (12.21)	21 (9.13)		174 (10.32)	8 (8.08)	

### Parameters associated with high blood pressure

The multivariate logistic regression for factors associated with high blood pressure is presented in Table 3. The three factors associated with high blood pressure in Model 1 and Model 2 were an increase in BMI z-score > 2, a decrease in HDL-C < 40 mg/dl, and an increase in LDL-C > 130 mg/dl. However, there was no association between average sleep duration and high blood pressure for either Group 1 or 3.

**Table 3:** Univariate and multivariate analyses of factors associated with high blood pressure

Parameters	All participants			Male participants			Female participants		
	**Univariate model OR (95% CI)**	**Multivariate model 1 OR (95% CI)**	**Multivariate model 2 OR (95% CI)**	**Univariate model OR (95% CI)**	**Multivariate model 1 OR (95% CI)**	**Multivariate model 2 OR (95% CI)**	**Univariate model OR (95% CI)**	**Multivariate model 1 OR (95% CI)**	**Multivariate model 2 OR (95% CI)**
BMI z-score ≤ 2	1	1	1	1	1	1	1	1	1
BMI z-score 2—< 3	**2.5 (1.4–4.4)**	**2.6 (1.3–5.2)**	**2.6 (1.3–5.2)**	**2.2 (1.4–3.6)**	**1.9 (1.1–3.3)**	**2 (1.14–3.4)**	**2.5 (1.4–4.4)**	**2.6 (1.3–5.1)**	**2.6 (1.3–5.1)**
BMI z-score 3—< 4	**3.9 (2.5–6)**	**4.7 (2.8–8)**	**4.7 (2.8–8)**	**3.7 (2.2–6.3)**	**3.1 (1.7–5.5)**	**3.1 (1.8–5.5)**	**2.6 (1.3–5.2)**	**3.4 (1.6–7.3)**	**3.4 (1.6–7)**
BMI z-score 4—< 5	**7.3 (4.2–12.5)**	**7.4 (3.9–14.3)**	**7.4 (3.9–14.3)**	**4.3 (2.3–8)**	**4.4 (2.3–8)**	**4.4 (2.2–8.8)**	**5.2 (2.4–10.9)**	**5.1 (2–13.1)**	**5 (1.9–12.8)**
BMI z-score 5—< 6	**10.7 (5.9–19)**	**12.1 (6.1–23.8)**	**12 (6–23.5)**	**6 (2.8–13.3)**	**8 (3.2–20.5)**	**8.5 (3.3–21.8)**	**4.5 (1.6–12.3)**	**7.1 (2.4–21.3)**	**6.8 (2.3–20.3)**
BMI z-score ≥ 6	**21.7 (12.2–38.8)**	**24.9 (12.6–49)**	**24 (12.6–49)**	**18.9 (8.9–39.8)**	**14.7 (5.8–36.9)**	**15 (5.9–37.5)**	**27 (13–55)**	**29 (12.7–66)**	**29 (12.8–68)**
HDL-C ≥ 40 mg/dl	1	1	1	1	1	1	1	1	1
HDL < 40 (each 5 mg/dl decrease)	**1.36 (1.17–1.57)**	**1.19 (1.0–1.4)**	**1.18(1.01–1.39)**	**1.3 (1.1–1.57)**	**1.26 (1.04–1.51)**	**1.25 (1.04–1.5)**	**1.40 (1.09–1.82)**	1.08 (0.78–1.5)	1.1 (0.78–1.49)
LDL-C ≤ 130 mg/dl	1	1	1	1	1	1	1	1	1
LDL < 130 (each 10 mg/dl increase)	**1.12 (1.02–1.22)**	**1.11 (1.0–1.23)**	**1.12 (1.02–1.23)**	**1.24 (1.1–1.37)**	**1.23 (1.1–1.39)**	**1.24 (1.1–1.14)**	0.97 (0.81–1.16)	0.93 (0.76–1.13)	0.93 (0.77–1.13)
TG ≤ 130 mg/dl	1	1	1	1	1	1	**1**	1	1
TG > 130 (each 10 mg/dl increase)	**1.2 (1.11–1.28)**	0.99 (0.9–1.09)	0.99 (0.9–1.09)	**1.2 (1.1–1.31)**	0.99 (0.89–1.1)	0.99 (0.89–1.1)	**1.01 (1.00–1.01)**	1.01 (0.86–1.19)	1.01 (0.85–1.19)
Average sleep Gr 2	1		1	1		1	1		1
Average sleep Gr 1	1.29 (0.99–1.69)		0.75 (0.54–1.03)	1.1 (0.78–1.48)		0.79 (0.54–1.18)	**1.7 (1.05–2.89)**		0.65 (0.31–1.15)
Average sleep Gr 3	0.95 (0.61–1.47)		0.81 (0.51–1.29)	0.76 (0.45–1.29)		0.89 (0.52–1.51)	1.26 (0.57–2.8)		0.61 (0.23–1.6)

*BMI* body mass index, *HDL-C* high-density lipoprotein cholesterol *LDL-C* low-density lipoprotein cholesterol, *TG* triglyceride Bold text, statistical significance (*p*-value ≤ 0.05)

## Discussion

The present study demonstrates that the prevalence of high blood pressure in adolescents aged 10–19 years in Thailand, based on the cutoff values from the 2017 AAP guidelines [1], was 9.4%. BMI z-score was independently associated with hypertension in a dose–response relationship for both genders and for all participants combined. However, the present study showed that neither short nor long sleep duration was associated with high blood pressure, even after adjustment for weekday or weekend variation.

This is the first study to represent the national prevalence of high blood pressure in Thai adolescents. The prevalence of high blood pressure in the present study was higher than in previous national-level studies conducted before the year 2017 in Iran, Australia and China, which reported the prevalence as 6.8%, 5.8% and 6%, respectively [18–20]. This finding could be partly explained by the fact that the cutoff values for diagnosis of hypertension of the 2017 AAP guidelines used in the present study were lower than those of the 2004 AAP guidelines [21] used in the studies from those countries. The 2017 normative blood pressure tables were derived from the 2004 normative blood pressure tables that did not include overweight or obese children and adolescents, and the cutoff values in the year 2017 guidelines were therefore 2–3 mmHg lower. Similar increases in the prevalence of hypertension resulting from use of these newer cutoff values have been reported in an international cohort and a study from Thailand [22, 23]. The prevalence of high blood pressure increased from 7% to 16.2% and from 6.9% to 10.8% in the international cohort and the Thai study, respectively.

The present study showed that in male participants, TC, LDL-C and TG levels were significantly higher and HDL-C levels were significantly lower in the high blood pressure group than the normotensive group. Among female participants, TG levels were significantly higher and HDL-C levels were significantly lower in the high blood pressure group than in the normotensive group. These results are consistent with the study reported by Garí-Llanes M et al., who found that among 373 children in Cuba aged 8–11 years, the high blood pressure group had higher levels of TC, LDL-C, TG and lower levels of HDL-C than those in the normotensive group [24]. In the present study, the higher BMI and greater proportion of obesity in the high blood pressure group could explain why they had higher levels of TC, LDL-C and TG and lower levels of HDL-C than did the normotensive group. In contrast, other reports from Serbia and Italy did not show a significant difference in those lipid profiles between children with high blood pressure and normotensive children [25, 26]. Because BMI and lipid profile are correlated in children and adolescents [27], the comparable BMI between the high blood pressure and normotensive groups in those reports from Italy and Serbia may explain why there was no difference in lipid profiles between the two groups.

Apart from the BMI z-score and lipid profile, sleep duration was found by previous studies to be another independent factor associated with high blood pressure [9–11]. The increases in sympathetic activity and appetite hormones such as ghrelin in individuals with short sleep duration have been proposed to be the mechanisms underlying this association [28]. Many studies have reported an association between sleep duration and blood pressure; Wells et al. found that short sleep duration was associated with increased SBP for both sexes in 4452 Brazilian adolescents aged 10–12 years [9]. Bal et al. studied 2860 Turkish adolescents aged 11–17 years and found that each hour of increase in sleep duration decreased the risk of hypertension by 12% [10]. The effect of an increase in sleep duration on a decrease in the risk of high blood pressure seemed to exist for both sexes. However, the present study did not show an association between sleep duration and high blood pressure. The discordance of the findings among these studies could be attributable to the divergent definitions of short sleep duration they used. To define a short sleep duration, Lee et al. used sleep duration ≤ 5 h/day in their study of 1187 adolescents aged 12–18 years [11], while Wells et al. used ≤ 8 h/day in adolescents aged 10–12 years and Bal et al. used the same definition in adolescents aged 11–17 years. In contrast, the present study defined the short and long sleep durations adjusted for age based on the recommendations of the NSF.

The present study represents the first identification of the national prevalence of high blood pressure in Thai adolescents. This study also demonstrated that BMI z-score as well as higher LDL-C and TG and a lower HDL-C were associated with high blood pressure after adjustment for the other parameters. Nonetheless, this study has some limitations. First, the cause-effect relationship between the risk factors and high blood pressure could not be determined from this cross-sectional survey. Second, high blood pressure was determined based on BP measurement at a single visit, not on three occasions as is recommended by the current guidelines; therefore a true diagnosis of hypertension could not be made in the context of this study. Data regarding awareness of hypertension, sleep-disordered breathing and co-morbidities were not available among these participants. Additionally, the sleep duration recommended by the NSF remains controversial, and therefore careful interpretation of the sleep duration results from the present study is warranted. Lastly, sleep duration was not measured using a validated self-reported tool specifically designed to measure adolescents’ sleep, such as the Adolescent Sleep–Wake Scale [29]. Therefore, more research on the association between sleep duration and the risk of high blood pressure in adolescents is needed.

## Conclusions

In conclusion, the overall prevalence of high blood pressure among adolescents in Thailand was 9.4%. BMI z-score was an important factor associated with high blood pressure. We found neither short nor long sleep duration to be associated with high blood pressure in the present study.

## Supplementary Information

**Additional file 1: Table S1. **The numbers of participants stratified by the sleep duration groups on** Table S2. **The numbers of participants with and without high blood pressure stratified by the sleep duration groups on weekdays and weekends

## Data Availability

The datasets used and/or analyzed during the current study are available from the Department of Community Medicine, Faculty of Medicine Ramathibodi Hospital, Mahidol University on reasonable request.

## Abbreviations

BMI: Body mass index
CI: Confidence interval
DBP: Diastolic blood pressure
HDL-C: High-density lipoprotein cholesterol
LDL-C: Low-density lipoprotein cholesterol
SBP: Systolic blood pressure
TC: Total cholesterol
TG: Triglyceride
WC: Waist circumference
WD: Weekdays
WE: Weekends
WHR: Waist to height ratio
