# Supplementary material for: Sleep duration and risk of high blood pressure in Thai adolescents: the Thai National Health Examination Survey V, 2014 (NHES-V)
Source: BMC Public Health. 2022 Oct 29;22:1983. doi: 10.1186/s12889-022-14430-z (PMC9617401; doi:10.1186/s12889-022-14430-z)
Supplement: Supplementary file 1 — Additional file 1: Table S1. The numbers of participants stratified by the sleep duration groups on Table S2. The numbers of participants with and without high blood pressure stratified by the sleep duration groups on weekdays and weekends [file 12889_2022_14430_MOESM1_ESM.docx]

**Table S1** The numbers of participants stratified by the sleep duration groups on weekdays and weekends

| **Sleep duration group** | **Weekend Gr. 1 (short)** | **Weekend Gr. 2**  **(normal)** | **Weekend Gr. 3**  **(long)** |
| --- | --- | --- | --- |
| **Weekday Gr. 1 (short)** | 448 (gr. A) | 441 (gr. B) | 101 (gr. C) |
| **Weekday Gr. 2 (normal)** | 157 (gr. D) | 1757 (gr. E) | 340 (gr. F) |
| **Weekday Gr. 3 (long)** | 4 (gr. G) | 51 (gr. H) | 206 (gr. I) |

**Table S2** The numbers of participants with and without high blood pressure stratified by the sleep duration groups on weekdays and weekends

| **Weekday-weekend group** | **Normotension** | **High blood pressure** |
| --- | --- | --- |
| A | 409 (91.3%) | 39 (8.7%) |
| B | 408 (92.5%) | 33 (7.5%) |
| C | 91 (90.1%) | 10 (9.9%) |
| D | 144 (91.8%) | 13 (8.2%) |
| E | 1570 (89.4%) | 187 (10.6%) |
| F | 310 (91.2%) | 30 (8.8%) |
| G | 4 (100%) | 0 (0%) |
| H | 47 (92.2%) | 4 (7.8%) |
| I | 193 (93.7%) | 13 (6.3%) |
| Total | 3176 | 329 |

**P* = 0.376
